# Experiences of Turkish nurses volunteering in the disaster zone following the 2023 Türkiye–Syria earthquake

**DOI:** 10.1111/inr.13056

**Published:** 2024-11-03

**Authors:** İnci Mercan Annak, Birgül Erdoğan, Nihal Yıldız Emre

**Affiliations:** ^1^ Faculty of Nursing, Department of Surgical Nursing Gazi University Ankara Türkiye; ^2^ Faculty of Health Science Department of Child Health and Diseases Nursing Kocaeli University Kocaeli Türkiye; ^3^ Faculty of Health Science Department of Surgical Nursing Kırıkkale University Kırıkkale Türkiye

**Keywords:** Disaster nursing, earthquake, emergency nursing, nursing, Türkiye–Syria earthquake

## Abstract

**Aim:**

The study aimed to determine the experiences of Turkish nurses who volunteered in the disaster zone following the 2023 Türkiye–Syria earthquake.

**Background:**

The earthquake devastated 11 cities in Türkiye, which required a comprehensive humanitarian response. Despite the crucial role of nurses in crisis management, limited research exists on their experiences in earthquake zones.

**Introduction:**

Nurses experience difficulties in disaster areas, such as physical conditions, lack of disaster plans, and inadequate disaster training before being deployed to earthquake areas.

**Methods:**

This study used a qualitative research model and case study design. Data were collected through in‐depth qualitative interviews. Nine participants who had worked in earthquake zones for at least 5 days were included in the study. The discussions were recorded, transcribed verbatim, and analyzed thematically. The purposive sampling method was used, and the Consolidated Criteria for Reporting Qualitative Research guidelines were followed.

**Results:**

As a result, 4 themes, namely physical conditions, psychosocial conditions, professional conditions, and organization, and their 14 subthemes were determined. This study revealed that nurses encountered difficulties related to physical conditions in earthquake zones. Moreover, the emotional burden expressed by the nurses highlighted the profound psychological impact of disaster response.

**Conclusion:**

This study determines that disaster preparedness and support for nurses should be enhanced to improve the arrangement of health services in future calamities.

**Implications for nursing policy:**

The results of this study can be used by nurse managers and healthcare policymakers to create nursing training programs that focus on disaster‐related competencies.

## INTRODUCTION

Natural disasters occur frequently worldwide and have significant and long‐lasting impacts on individuals, families, communities, and the environment (Li et al., 2017). Earthquakes are typically more deadly than other types of natural disasters. On February 6, 2023, two earthquakes of magnitudes 7.7 and 7.6 occurred in Türkiye, 9 h apart, with epicenters in the Pazarcık and Elbistan districts of Kahramanmaraş, respectively. As the consecutive occurrence of two earthquakes of this magnitude affected a large geographical area over both Türkiye and neighboring Syria, this disaster was referred to in the literature as the 2023 Türkiye–Syria earthquake (Naddaf, 2023). This was one of the largest earthquakes never experienced before and is referred to as the catastrophe of the century. The earthquake devastated 11 cities in Türkiye, including Kahramanmaraş, Gaziantep, Şanlıurfa, Diyarbakır, Adana, Adıyaman, Osmaniye, Hatay, Kilis, Malatya, and Elazığ, which required a comprehensive humanitarian response. At least 45,089 people lost their lives as a result of the earthquake, with thousands injured and millions left homeless (AFAD, 2023a).

A total of 332 tent cities, 360,167 tents and 89 container cities were set up in the earthquake‐affected region. A total of 1,440,668 people in tents and 34,120 people in containers were received shelter services. (AFAD, 2023b). In addition to the basic needs of the citizens living in the earthquake zone, such as shelter, food, and clothing, demand for health and treatment services increased (Rezaei et&amp;#x000A0;al., 2020).

As part of this process, the Ministry of Health created an application form through the “EKİP Portal Application” for doctors, nurses, and healthcare professionals who wished to volunteer in the disaster area to submit their requests. The volunteer applications were reviewed by the relevant provincial directorate of health, and the Ministry of Health assigned them to the region in line with the needs. These assignment requests were received through the personnel information system (Saglık Bakanlıgı, 2023). Nurses are not only victims but also rescuers in a major disaster. Therefore, they play a key role in managing the consequences of natural disasters. Nurses as rescuers play a critical role in disaster relief after the earthquake. They often work on the front lines of care and are the group of healthcare professionals most needed in disasters (Liao et al., 2019; Rezaei et al., 2020).

The recent earthquake in Türkiye revealed significant weaknesses in our institutional and personal preparedness for such emergencies. Particularly in the earthquake‐affected region, deficiencies in health services and health management and care disruptions underscore the need for strengthening in the earthquake area. In the aftermath of an earthquake, there is a marked increase in the physical, emotional, mental, and social demands of the population, with corresponding peaks in demand for health and treatment services (Liao et al., 2019; Moradi et al., et al., 2020). Nurses, in particular, have roles beyond simple clinical care during a crisis, with expanded responsibilities in crisis management (Nakayama et al., 2019). They can provide care before, during, and after crises thanks to their critical clinical and administrative skills, reducing injury mortality by 50%–70% (Firouzkouhi et al., 2021). Even though nurses are known for their quick thinking, effective communication, and creative problem‐solving in emergencies, there are still ongoing gaps in their preparedness training that can be filled to enhance nurses' proficiency in managing all types of disasters (Segev et al., 2024). In such a situation, they must assess individual and collective needs, evaluate the physical, mental, spiritual, cultural, and ethical status of individuals and families, and strategically plan their responses (Rezaei et al., 2020).

In large‐scale disasters, nurses often serve as first responders, yet many feel inadequately equipped to work with limited resources in such situations (Li et al., 2017). Studies emphasize not only the need for disaster management training but also the importance of nurses' psychological well‐being when working in disaster‐stricken areas (Li et al., 2015; Li et al., 2017; Moradi et al., 2020; Yan et al., 2015; Yin et al., 2011). To facilitate substantial investment in preparing nurses for potential disasters, a comprehensive understanding of their roles, needs, and capabilities in disaster contexts is essential.

The continued relevance of earthquake disasters and the inherent regional seismic risk of Türkiye further emphasize the importance of this topic. Despite the crucial role of nurses in crisis management, limited research exists on their experiences in earthquake zones (Li et al., 2017; Moradi et al., 2020; Rezaei et al., 2020). Among the research on the earthquake that struck Turkey and Syria, none that looked at the experiences of volunteer nurses who went to the earthquake zone were found in the literature review (Harmanci Seren & Dikeç, 2023; Kaya & Erdogan, 2024; Sert et al., 2023). In this regard, our study is the first to focus on volunteer nurses' experiences during the earthquake in Turkey and Syria. Moreover, it focuses on the experiences of volunteer nurses in field hospitals in different earthquake regions, giving voice to these volunteers and describing professional competencies and conditions essential for them to provide quality care.

## METHOD

In this part of the study, detailed information is provided in the research model and design, research team, data collection tools, implementation of the research, participants, data analysis, validity and reliability, and ethical aspects of the research sections.

### Research model and design

This study utilized a qualitative research model. As Creswell states, qualitative research begins with certain assumptions and utilizes interpretive/theoretical frameworks that guide the investigation of research problems, focusing on the meanings that individuals or groups ascribe to social or human issues. In qualitative research, a researcher seeks to elucidate and understand concepts, phenomena, and relationships through interviews, conversations, photographs, and recordings. The collected data are then coded and categorized, and the research findings are presented based on these codes and categories (Creswell, 2013).

The qualitative research model can incorporate various research designs, and in this particular study, the case study design was used. Creswell characterized a case study as a qualitative research approach, which allows a researcher to delve into one or more time‐bound situations using various data collection tools, such as observations, interviews, audio–visuals, documents, and reports, and then outline situations and themes relevant to the case (Creswell, 2013). Yin further described the case study as a research method particularly suited to answering “how” and “why” questions, particularly when the researcher has little or no control over the events under investigation, when the phenomenon is studied in its natural context, and when the connection between the event and real life is somewhat ambiguous (Yin, 1984). As the study was conducted within the framework of a specific situation, the “purposive sampling” method was used. Thus, researchers aim to obtain a sample that best represents the situation they want to analyze. In case studies, when the researcher wants to examine a specific event, it is important to select a sample that represents relevant situations (Beycioğlu et al., 2018). In our study, the “case” is the earthquake disaster. All participants in our study were selected through purposive sampling from nurses who had worked at least 5 days in field hospitals in different parts of the earthquake zone.

### Research team

The research team consists of 3 academic nurses. The first researcher holds a Ph.D. in Surgical Disease Nursing and serves as a lecturer in this discipline. The second researcher is a Ph.D. candidate in Pediatric Nursing and holds the position of research assistant. Similarly, the third researcher is completing her Ph.D. in surgical nursing and is also a research assistant. All researchers have previous training and experience in qualitative research methods. The third researcher (NYE) conducted in‐depth qualitative interviews, while two researchers (BE and NYE) were responsible for primary and secondary coding. Each researcher played a role in transcribing the data (IMA, BE, and NYE), analyzing the data, and developing research themes (IMA, BE, and NYE). Within the participant group, two individuals were personally known to the researchers, with no relationship between the researchers and the remaining participants. At the beginning of the interviews, the participants were provided with general information about the researchers, such as their professional experience and educational background.

### Data collection tools

The data of the study were collected using a data collection form prepared by the researchers based on existing literature (Liao et al., 2019; Moradi et al., 2020; Nakayama et al., 2019; Rezaei et al., 2020). The data collection tool comprises two forms: personal information form and semi‐structured interview form.

*The personal information form* includes eight questions related to gender, age, educational background, professional tenure, training related to disaster nursing, time of appointment to the region, specific disaster area, and duration of appointment.

*The semi‐structured interview form* was formulated by the researchers to capture the experiences of nurses working in field hospitals in earthquake zones. The questions in this form were prepared by the researchers considering the information they obtained from their colleagues, social media, news, and other information sources (professional organizations, etc.) that they had been following since the beginning of the disaster during the earthquake period. This form includes two questions designed to explore the experiences of nurses and suggestions.

1. What were your experiences while volunteering at the field hospital in the earthquake zone?
2. Physical conditions (hygiene, access to food, shelter, and risk or experience of epidemics)
3. Psychological status (related to patients receiving care and destruction in the earthquake area)
4. Social relationships (inability to communicate with family, friends in the workplace, and loneliness)
5. Based on your experience at the field hospital, what would you recommend to nurses in a disaster area?
6. Recommendations for individuals who will volunteer to go to the earthquake zone
7. Recommendations for institutions that will send their personnel to the earthquake zone
8. Recommendations for the government/ministry

### Implementation of the research

The study was conducted in 2023 with nurses who volunteered their services in field hospitals in the earthquake zone. Research data collection began in March 2023, with nurses appointed to the area who volunteered to participate in the study. Subsequently, the snowball sampling method was implemented, resulting in the continuous recruitment of participants until data saturation was reached.

In this study, purposive sampling was used to select participants. According to this method, eligible nurses had to have worked for at least 5 days in one of the field hospitals established in the region and voluntarily agreed to participate in the study. After the disaster, the Ministry of Health assigned nurses to field hospitals in the region for shorter periods (such as 5 days) in the acute phase after the earthquake and for longer periods (such as at least 10 days) in the following days. The assignment was entirely voluntary. This is why nurses who worked in the region for at least 5 days were included in the sample. Nurses were introduced to the study in person and through online sharing platforms (e.g., WhatsApp and Instagram) and invited to participate. Once the nurses who agreed to participate completed the informed consent form, the research team explained the study objectives and data collection forms to them and answered any questions. In‐depth qualitative interviews were then conducted using an online interview platform and recorded with participant consent. These interviews ranged in length from 20 to 43 min.

All interviews were conducted by the researcher (RN), who had previous experience with in‐depth individual interviews and qualitative research. The interviews were confidential to the researcher and the individual participants. After transcription, the interviews were shared with the participants to obtain their consent. The data collection process was completed when the qualitative data reached saturation.

### Participants

The study included a total of nine participants. Among them, three were male and six were female, with ages ranging from 26 to 49 years. In terms of educational background, five participants had a bachelor's degree in nursing education, while the remaining four had a master's degree. Their work experience ranged from 3 to 10 years. Four participants reported that they had previously received training in disaster nursing practice.

Participants traveled to the affected region between 1 and 60 days after the earthquake and volunteered at field hospitals for 5–33 days. As the earthquake affected 10 provinces, the volunteer nurses were sent to different locations. Specifically, the study participants were assigned to Hatay (including the center, İskenderun, Serinyol, and Antakya), Kahramanmaraş (center and Göksun), Şanlıurfa (center), and Adıyaman (Kahta). As these regions were the most affected, our participants reflect the general view of the phenomenon.

### Data analysis

The research was reported using the Consolidated Criteria for Reporting Qualitative Research (Tong et al., 2007). The data were analyzed using qualitative content analysis methods, a dynamic approach to analysis that makes use of all available data and involves simultaneous collection and analysis (Sandelowski, 2000). Content analysis in the processing of qualitative research data is typically conducted in four stages: (1) data coding, (2) theme identification, (3) organization of codes and themes, and (4) definition and interpretation of findings (Yıldırım & Şimşek, 2006).

In this study, the analysis and interpretation of the collected data were carried out in the following stages:

- Participants were each assigned a unique number to protect their information.
- Three researchers (IMA, BE, and NYE) independently completed the data analysis process by repeatedly reading the material until they were thoroughly familiar with it.
- After analysis, codes were assigned to the main thoughts and ideas from the data.
- These codes were then structured into themes and categories that adequately represented the data being analyzed.
- The participants' views were carefully evaluated throughout the analysis process, and the codes were linked to the emerging themes and categories.
- After the themes were created, they were shared with all participants, and their opinions were asked. The participants did not request any changes. Accordingly, the final version of the themes was given.
- Code frequencies were calculated; data were interpreted; the results of the study were documented to determine the validity and reliability of the study.

If the data analysis is to be exemplified, the participants mentioned that toilets were a big problem because they could not find showers and bathrooms in the area. Key ideas such as shower, bathroom, toilet, and smell were coded here. These codes were then interpreted by the researchers and combined under the hygiene subtheme. The same process was followed for subthemes such as nutrition, housing, and security. Looking at the subthemes, all of these factors were related to physical conditions, and the physical conditions theme was created.

### Validity and reliability

Scientific research must demonstrate a certain level of validity and reliability to be accepted. To achieve this, a sample of sufficient size must be selected; the data must be reviewed by more than one researcher; additional sources and opinions must be consulted; careful coding must be applied to the data obtained; each step of the data analysis process must be detailed; the research environment and the researcher's role must be fully described in the research report; and the study must be conducted impartially without the influence of the researcher's personal views (Baltacı, 2018; Denzin & Lincoln, 2008; Elo et al., 2014; Hruschka et al., 2004).

Credibility was increased by sharing the transcribed text with participants after the interview to obtain their consent and by independent data analysis by three researchers (IMA, BE, and NYE). Transferability was enhanced by clearly describing the data collection process, providing essential descriptive information about the participants, and elaborating on the data analysis process. The reliability of the findings was strengthened by sharing the researcher's themes and categories with participants to obtain their perspectives and by using quotes to support each theme and category. Confirmability was enhanced by having two researchers analyze the data and repeating the coding line‐by‐line. The originality of the study was enhanced by allowing participants to openly express their opinions on all topics and using purposive sampling.

### FINDINGS

An analysis of the sociodemographic characteristics of the nurses who participated in the study revealed that there were six female and three male nurses. The majority of the nurses, seven individuals, had more than 5 years of work experience. Educational backgrounds were distributed between bachelor's and master's degrees, with five nurses having a bachelor's degree and four having a master's degree. The nurses were assigned to field hospitals in locations such as Hatay, Adıyaman, Kahramanmaraş, and Şanlıurfa, with the majority stationed in Kahramanmaraş, the epicenter of the earthquake, and Hatay, where the destruction was the most extensive. Of the nurses working in the field hospitals, only three (H2, H5, and H8) received special training on disaster nursing; the rest did not receive any training (Table 1). The mean age of the nurses was 31.88 ± 7.60 years, and the mean length of service in the field hospitals was 11.22 ± 8.7 days.

**TABLE 1:** Sociodemographic characteristics of the participants.

Nurse no.	Age	Gender	Education status	Professional experience	Place of duty	Duration of stay
H1	30	Male	Master's degree	10 years	Hatay	10 days
H2	30	Male	Bachelor's degree	7 years	Adiyaman	7 days
H3	49	Female	Bachelor's degree	9 years	Kahramanmaraş	8 days
H4	40	Female	Bachelor's degree	17 years	Kahramanmaraş	9 days
H5	29	Female	Master's degree	10 years	Şanliurfa	33 days
H6	27	Male	Master's degree	4 years	Kahramanmaraş	15 days
H7	28	Female	Bachelor's degree	6 years	Hatay	9 days
H8	26	Female	Bachelor's degree	2.5 years	Hatay	5 days
H9	28	Female	Master's degree	6 years	Hatay	5 days

As a result of the content analysis, four themes, namely physical conditions, psychosocial conditions, professional conditions, and organization, and 14 subthemes belonging to these themes were identified. The themes and subthemes of the research are shown in Table 2.

**TABLE 2:** Experiences of nurses working voluntarily in the disaster area—Themes and subthemes.

Themes	Subthemes
Physical Conditions	Hygiene
	Nutrition
	Housing
	Security
	Environment
	Epidemics
Psychosocial Conditions	Emotions
	Sociability
Professional Conditions	Patients/cases
	Professional Competence
	Working Conditions
Organization	Individual Organization
	Corporate Organization
	Organization of Earthquake Survivor Nurses

### Theme 1: Physical Conditions

Participants shared their experiences of the physical conditions in the earthquake zone they visited as volunteers under six subthemes: hygiene, nutrition, housing, security, environment, and epidemics.

**Hygiene**: Participants in the study were consistent in reporting problems with hygiene in their work areas and in sharing negative experiences. In the aftermath of the earthquake, factors such as limited toilet and shower facilities, inadequate water supply, cold weather, and lack of hot water posed significant challenges to maintaining personal hygiene. One participant described the situation this way:

“There were 500 medical personnel there, and a bathroom was brought in the form of a container. There were three shower rooms for 500 people in that container. Of course, there were private rooms inside the hospital where we could shower, but because it was an earthquake zone and there were still aftershocks, people were afraid to go inside the building. We had to wait in line for the bathroom in this container.” (H1)

“Sanitation conditions were poor. Families were staying together because they were afraid to enter their homes, which led to an increase in diseases such as influenza and rotavirus, which increased rapidly, especially in public places. Especially respiratory diseases were more common.” (H6)

One nurse described how they worked together because the number of bathrooms was insufficient. She said:

“For example, there were soldiers who came to set up tents. They also have personal needs. People have to complement each other's needs. We said, ‘There is hot water in our room; you can use it if you want’. We tried to help some people.” (H9)

Another nurse used the following expressions when describing hygiene problems:

“We did not have shower facilities when we stayed in the tent. The water was already cut off in the first period, and there was no mains water. We were staying in tents in the garden of a school. We could meet our toilet needs by using the school toilet. We met our needs with transportation and water. We had a lot of difficulty in meeting our needs in terms of cleaning.” (H5)

Another participant indicated that he did not care much about the hygiene deficiencies:

“When you go there, you do not think about comfort, hygiene, nothing. You just think and plan to help the people there. I did not care about hygiene or hunger. The goal was to hold someone's hand.” (H3)

**Nutrition**: The nurses who participated in the study stated that there were problems finding food and clean water in the first few days after the earthquake and that food and water were distributed by NGOs, philanthropists, and official agencies on the following days. Some nurses said they cooked their own meals where they were told to stay. One nurse said:

“We were a bit luckier. We cooked and cleaned with our own means, but the conditions were really hard and terrible. We were prepared, and since there was a kindergarten in our neighborhood, we had food supplies.” (H3)

Another nurse expressed the situation in her region in the following way:

“We had soup and bread every day, no meals. One day, when it was a holy day, there was a meat meal. For 5 days we ate soup and bread. We had brought some things with us and ate them, so we had no problems. Housing, catering and drinking were difficult but not too difficult; after all, we were there for a purpose.” (H8)

**Housing**: Nurses typically chose containers or tents because the buildings were unsafe due to damage and ongoing aftershocks. The cold winter weather was a significant challenge. One nurse spoke of the tangible effects of the aftershocks, saying “We were particularly affected by the aftershocks in the region. We could feel the aftershocks with the shaking of some materials like IV poles. Once I felt the ground move even though I was in the tent. However, there was nothing to collapse, so we were not that scared.” (H5)

Another nurse said, “The first time we went there, there was a school right next to the field hospital”. People had turned the school into an operating room. They told us we could stay here, but we did not want to stay in the building because it was an earthquake zone and there were big tremors. Thus, they told us, “You can move around by yourself, you can stay wherever you want to stay, so we found an empty container and stayed there.” (H1)

Security: Some nurses took responsibility for safeguarding their belongings as rooms did not always have lockable doors. One nurse expressed uncertainty about the security of personal items: “Not every room had a key to lock the doors. Our room was not a lockable room. We would take our personal belongings and go to work. In addition, we were never sure there would be visitors when we were not in the room.” (H8)

Despite communal living, most nurses did not perceive security risks, though some expressed reservations about staying in tents.

**Environment**: The region was littered with debris from the destruction, and harsh weather conditions exacerbated the environmental challenges. Participants remarked on the following issues:

“Since some people lived in tents all the time, it was inevitable that the inside would get muddy even with a little rain or rainfall.” (H5)

“There were power outages, dust and smoke, and lots of tremors.” (H9)

**Epidemics**: In the aftermath of the earthquake, due to continuous aftershocks and the lack of structurally sound buildings, people from the affected region, as well as outside aid workers, were relocated to communal living areas. This arrangement was particularly conducive to the outbreak of epidemics. In addition, some epidemics were attributed to the consumption of non‐potable water. Nurses shared their experiences on these issues:

“Upper respiratory tract infections and acute gastroenteritis were widespread in young children.” (H5)

“Hygiene conditions were poor. Families were afraid to enter their homes; thus, they either stayed in another neighborhood or stayed together, which led to an increase in diseases such as influenza and rotavirus, which increased rapidly, particularly in communal settings. In particular, respiratory diseases were more common” (H6).

### Theme 2: Psychosocial Conditions

Participants shared their perspectives on their psychosocial experiences in the earthquake‐affected area, categorizing them into two subthemes: emotions and sociability.

**Emotions**: The nurses who participated in the study unanimously described the emotional challenges of being in the affected region. However, the magnitude of the disaster, the vulnerability of the affected population, and the urgent need for assistance prompted the nurses to subordinate their emotional states and assist those in dire need. The most prominent emotions were empathy, helplessness, exhaustion, pain, motivation, and determination. One nurse articulated her motivation as follows:

“Seeing the desperation of the people there made me stronger. Because they were already broken, and we could not recover if we were broken too.” (H3)

Another nurse who volunteered three times in the earthquake zone described how she kept herself motivated:

“Therefore, I can say that it changed my perspective on life. Maybe the reason I went the third time was because I wanted to do something. Maybe not big things, but I wanted to touch their hearts, communicate with them and make them feel really happy. Maybe even playing a game with a child or listening to someone's problems made them feel that we were with them and the spiritual dimension was very real.” (H5)

Regarding the psychological impact of the earthquake, another participant said “You know, psychology is like an epidemic, it affects each other. Someone else's anxiety, and depression infects you. Of course, their bad mood, their anxiety about the possibility of another earthquake reflected on us.” (H6)

Some nurses’ descriptions of the emotional impact of caring for the children of earthquake victims are as follows:

“Especially children were more affected than adults. These children's cases affected us more.” (H2)

**Sociability**: Despite communication challenges in the affected region, such as limited opportunities for family contact, the participating nurses were able to foster socialization within their team thanks to the workload. This dynamic prevented feelings of loneliness among team members. Participants' comments on this issue were as follows:

“It did not affect me much socially. The people there needed me more than my family.” (H1)

“We had no social problems. Everyone was helpful and cooperative.” (H2)

“I was the only one from my institution who went to the region, but I didn't feel lonely because all the friends who went were there for a purpose. When the goal was united, it was easier to stick together.” (H3)

A nurse's statement about her experience with the team she worked with in the earthquake zone is as follows:

“There was a WhatsApp group of the Emergency Team. The nurse in charge said, ‘Do not leave the group and stay’, and ‘I do not know when it will happen, but when Hatay returns to its good old days, I will host you well’. We are still in social contact with her.” (H9)

### Theme 3: Professional Conditions

Participants articulated their experiences of the professional environment in the field hospitals where they were stationed, categorizing their insights into three distinct subthemes: patients/cases, professional competence, and working conditions.

**Patients/cases**: In the earthquake region, the presence of amputations, sequelae, and severe trauma were mentioned. Caring for children and the elderly became particularly emphasized. Many nurses stated that they had seen cases they had not seen before and drew attention to the psychology of earthquake victims. One nurse said, “The psychology of the people there may not be able to deal with a lot of things. Because of fear and anxiety, people could easily loot the aid that arrived.” (H3)

A nurse caring for a child patient said, “For example, there was a boy. We gave him a toy car, and on the box was a stick to drive the car. But the stick was not in any of the boxes. The child was in tears for a stick until morning. Finally, we got together with our nurse friends. We made something like a stick with syringes and attached it to the car. That was the only way the child did not cry. It was good, and I was very psychologically affected.” (H8)

**Professional Competence**: The nurses who participated in the study indicated that age, experience, education, and expertise were important in the earthquake zone. The participants' statements on this issue are as follows:

“Especially new graduates and newly appointed friends may be very eager to go, but I do not recommend them to go.” (H1)

“First of all, our colleagues who will go to the region should definitely be experienced in this field and receive training on nursing care in disasters.” (H2)

**Working Conditions**: Participants described the challenges, including extended working hours, fatigue, sleeplessness, overwork, lack of supplies, and orientation problems. They expressed that their responsibilities extended beyond traditional nursing services to include many other tasks in the earthquake zone. One nurse's statement on these challenges is as follows:

“Especially in such a devastating disaster, there comes a time when you are not just a nurse; you take on every role. I saw the director of health carrying a bed on his back; he did not say he was the director. You have to make soup for people. For example, we had a senior nurse; on the third day, she was giving an injection, and somebody stopped her and said ‘What are you doing? Aren't you a cook?’ She said ‘No, I'm a midwife’, because for three days, she only cooked for the staff at the school.” (H4).

### Theme 4: Organization

Participants shared their insights about organizations in the areas where they worked after the earthquake, and these insights were categorized into three subthemes: individual organization, corporate organization, and organization of earthquake survivor nurses.

**Individual Organization**: Participants offered personal recommendations for nurses who may be appointed to earthquake zones, emphasizing the importance of preparing individual earthquake bags and participating in emergency training. One participant's statement in this regard is as follows:

“First of all, everyone should have their own personal disaster kit. They should be able to take care of themselves to provide an effective and healthy service to the other party. Our own mental health is also essential. Maybe we can bring a favorite food or food to motivate us.” (H5)

**Corporate Organization**: The participants' expectations from the institution are as follows: to provide in‐service and emergency training, create volunteer lists, ensure the organization of nurses in the earthquake zone, and improve the working hours and remuneration of nurses working in the earthquake zone. The participants' statements in this regard are as follows:

“For example, the training nurses could have given a training because it was clear that we were going as volunteers. Therefore, they could have at least given training on what to do in case of a disaster. I have a master's degree, but I never worked in a disaster area.” (H1)

“The people who went there were not categorized in any way. In order to make the best use of that person, it is necessary to use him or her in his or her own field.” (H2)

“First of all, they have to meet the housing needs of the people, they have to ensure their safety, and of course, to motivate them; you have to give them compensation that satisfies them in those regions.” (H4)

**Organization of Earthquake Survivor Nurses**: The participating nurses discussed the issues of leave entitlement and psychosocial support for their colleagues who were also earthquake survivors and continued to work in the affected area. They revealed that some of the health workers who survived the earthquake did not want to stay in the affected area and instead chose to live in different cities and commute to the area only for their shifts. The participants' statements on this matter are as follows:

“I think the people there need most right now is psychosocial support. Everyone there, from the most junior health worker to a professor with the highest title, needs psychosocial support. They need to realize that as soon as possible.” (H2)

“They should not force earthquake victims who do not want to work there. When I was there, many people from Istanbul and Kayseri went to the region this way. I think they can be provided with some relief.” (H6)

## DISCUSSION

This study examined the experiences of volunteer nurses who assisted in field hospitals established in regions affected by a major earthquake disaster that affected 11 cities. Semi‐structured interviews were used to collect data, and four themes were identified. The analysis of these nurses' experiences will help focus on the needs perceived by those providing care in the aftermath of an earthquake, thereby aiding in the development of tailored interventions and programs and enhancing preparedness for analogous acute situations.

In reviewing the existing literature, most studies on nursing practice during earthquakes have typically focused on aspects such as obstacles faced by teams en route to the affected area, costs of inappropriate disaster health services, lack of disaster plans, and inadequate disaster education for nurses deployed to the earthquake zone (Kalanlar, 2019; Li et al., 2017; Moradi et al., 2020; Nakayama et al., 2019; Rezaei et al., 2020).

In this study, the majority of nurses working in field hospitals did not have any specific training or coursework related to disaster nursing. Li et al. (2017) reported that although all participants had a wide range of clinical experience, nearly half had received disaster training, and about a quarter had prior experience in earthquake response. According to Abdi et al. (2021), participants claimed that nurses were unable to fully utilize their skills during a crisis due to the lack of specialized disaster training programs. Another study involving nurses working in earthquake zones emphasized that basic disaster education should be extended to nursing students and clinical practitioners (Whetzel et al., 2013). In our study, there was a unanimous request from all nurses for continuing education in disaster management.

Fostering an educational culture that promotes disaster preparedness and awareness among all healthcare teams, with a special focus on nurses, is paramount. Nurses need to be equipped with basic skills in health promotion, risk reduction, and disease prevention during disasters. Accurate, timely, and appropriate nursing care is critical to ensuring survival, reducing mortality, and improving well‐being after disasters. Therefore, through specialized disaster education and training programs, nurses can develop professional and technical skills, such as the use of emergency equipment, triage, and providing psychological care to victims, even before a disaster strikes. Integrating initial disaster nursing skills into undergraduate education and formulating control units to provide specialized training is critical.

The themes identified in this study revealed that nurses encountered difficulties related to hygiene, nutrition, housing, safety, environment, and epidemics related to the physical conditions in the earthquake zone. They noted that nutrition‐related issues in the acute phase were alleviated by distributions from nonprofit and government agencies in subsequent periods. However, challenges related to hygiene, housing, security, environment, and epidemics did not diminish over time but rather intensified. Existing studies also confirm the existence of unfavorable physical conditions for nurses after earthquakes (Li et al., 2017; Nakayama et al., 2019; Yan et al., 2015). It is hypothesized that nurses' negative experiences related to these physical conditions not only negatively affect their personal health but also their motivation to work in field hospitals. This scenario further exacerbates existing challenges in working conditions and limits opportunities for rest, culminating in physical and psychological fatigue among nurses. Therefore, it is essential to address these challenges through appropriate policies, planning, and support mechanisms to improve the well‐being of healthcare providers and the quality of care provided. However, views may differ on this issue, and further research could provide additional insight into these issues.

The emotional burden and psychological fatigue expressed by nurses working in the earthquake zone are consistent across studies, highlighting the profound psychological impact of disaster response. All the nurses who took part in the research reported experiencing a lot of pain, emotional exhaustion, and helplessness, but they were forced to suppress their own emotions due to the severity of the earthquake and the victims' helplessness. This circumstance highlighted the nurses' motivation, empathy, and determination. Similar to our study, Yan et al. (2015) reported that nurses working after the Wenchuan earthquake experienced psychological problems. The same study also found that no research has attempted to identify and quantify the total number of nurses across China who suffered from long‐term stress and trauma effects after this earthquake (Yan et al., 2015). Salmani et al. stated that the potential problems and physical and mental conditions of volunteers working during and after disasters should be evaluated (Salmani et al., 2019). Another earthquake study stated that one of the main elements of nursing services when facing disasters was providing psychological support (Hugelius & Adolfsson, 2019). In addition, there are ethical issues such as nurses taking too much responsibility for patients, sometimes making decisions for them, and having to choose among patients. These situations show that nurses get involved in ethical dilemmas and have to carry the emotional burden of their decisions. In our study, most nurses mentioned this emotional burden and reported feeling guilty, inadequate, and frustrated, even when they left the region. The findings of Moradi et al. are similar to our results (Moradi et al., 2020). While post‐disaster conditions are rapidly changing, one of the most critical issues to emphasize is the psychosocial status of nurses. It is believed that since they lack experience, education, and the capacity to control chaotic and stressful conditions, nurses working in earthquake zones suffer more significant levels of negative emotions during disaster events. Nurses need to be psychologically strong enough to perform their roles effectively in the disaster area. Nurse leaders and health policy makers should plan training to increase their emotional resilience and educate them on handling emergencies. As a result, nurses will provide better treatment and be more prepared to act quickly in emergencies, such as earthquakes. Ensuring and supporting the psychological resilience of nurses should be organized by the nursing organization because providing psychological support in the face of disaster is one of the basic elements of nursing services.

Another theme of the study was experiences related to professional conditions. In this theme, participants reported that they faced patients in the field hospital that they had not encountered before, did not have sufficient professional competence to care for and treat these patients, and the working conditions were very harsh. Participants reported problems related to staffing, material support, reserves and storage, and management of relief supplies in the study by Li et al. (2017). Other studies (Abdi et al., 2021; Nakayama et al., 2019; Yan et al., 2015) concluded that nurses commonly experience material and human resource shortages during disasters, in addition to problems such as excessive workload and long working hours. The infrastructure problems and working conditions were exacerbated by the massive destruction of the Türkiye–Syria earthquake, known as the disaster of the century. This study highlights the value of administrative research in evaluating nurses' working conditions during emergencies like earthquakes. This situation demonstrates the importance of establishing a sound system for communicating and sharing information between disaster relief teams and local hospitals. Disaster training programs are advised to incorporate the nursing experiences identified in this study. Disaster management and care courses should be offered in undergraduate and graduate nursing programs at nursing schools and in‐service training in hospitals. It is recommended that simulation techniques be employed in planning of these trainings.

In the final theme of the study, nurses shared their experiences of organizational inadequacies. Abdi et al (2021) reported findings that were consistent with the results of our study. Our study found that nurses experienced confusion due to unclear role definitions, uncertainty about their responsibilities, and coordination challenges due to inadequate institutional organization. Pouraghaei et al.’s study following the Azerbaijan earthquake revealed that the lack of inter‐organizational coordination was a key challenge (Pouraghaei et al., 2017). This underscores the need for a cohesive system that requires organizations to respond to massive disasters, such as earthquakes. This system must include training nurses on accessible resources (e.g., relief supplies), stress management, and counseling services for survivors, communities, and health professionals, and follow health management principles throughout the prevention/mitigation, preparedness, response, and recovery/rehabilitation phases. Establishing an effective communication and information‐sharing system between disaster response teams and local hospitals is also critical, among other areas suggested for disaster training.

It is recommended to establish disaster nursing programs in postgraduate education in our country, thereby addressing the aforementioned issues by designing scenarios based on the experiences of nurses.

## CONCLUSION

In the study, nurses' perceptions about their transfer from hospitals to the disaster area, their arrival to the affected area, their efforts to work in field hospitals, and the difficulties they encountered were investigated. Although most participants had experience working in hospitals, some issues emerged as necessary in this research. These problems include nurses' concerns about disaster preparedness, confusion about roles in the disaster area, and uncertainties about disaster nursing roles and responsibilities. These challenges underscore the need for professional and individual education focused on appropriate disaster response and the imperative to take urgent institutional action and develop an effective disaster strategy. The data collected is expected to form the basis for formulating policies regarding disaster nursing education and training, thereby increasing the ability of nurses to best prepare for their interventions in disaster‐affected areas.

The data obtained from the nurses participating in the study provide valuable results for structuring health services in the region and arranging nursing care, particularly in disaster situations. The number of samples in our research includes nurses that the researchers can reach individually and is limited. In this context, it is crucial for health policy makers to reach all nurses who work in the region and get more opinions from a larger group. Different opinions and experiences will benefit the education sector and the policies to be developed.

### Limitations

Despite the wealth of empirical findings derived from the results of the study, a number of limitations must be acknowledged. First, the information obtained focuses exclusively on the experience of the 2023 Türkiye–Syria earthquake, limiting its broader applicability. Second, the findings were limited to the relatively short time that the participants spent in the earthquake‐affected area, as shared through the conduit of our research, further limiting the scope of the conclusions that can be drawn. Third, this study produced rich data; the study's sample size may be one of the limitations.

## IMPLICATIONS FOR NURSING AND HEALTH POLICY

According to these results, health institutions and schools, politicians, and nurses have essential roles. Health institutions should support nurses working in their units with emergency action plans, earthquake drills, and in‐service disaster training for disaster situations. In addition, a disaster response team should be formed according to the individual characteristics, experience, and expertise of the employees and strengthen the first response group in the case of disaster. Nursing schools should include disaster management and care courses in undergraduate and graduate nursing education and conduct drills, on‐site observations, and role‐play studies (on amputee patient care, emergency care, perioperative care, and crush care). Politicians should provide the necessary financial support for educational activities. Politicians are also responsible for motivating nurses working in the disaster area with additional wages and providing psychological support. On the other hand, nurses should be involved in in‐service training on disasters and strengthen their knowledge and equipment on emergency care. They should also improve themselves in communication, coordination, leadership, and cooperation.

It is our belief that coordinated efforts between nurse leaders and other leaders in the health, education, and government sectors in Türkiye are desperately needed to guarantee that nurses are equipped to provide frontline care during emergencies.

## AUTHOR CONTRIBUTIONS

Study concept and design: İnci MERCAN ANNAK and Birgül ERDOĞAN. Collection of data: Nihal YILDIZ EMRE. Analysis and interpretation of data: İnci MERCAN ANNAK, Birgül ERDOĞAN, Nihal YILDIZ EMRE. Drafting of the manuscript: İnci MERCAN ANNAK, Birgül ERDOĞAN, Nihal YILDIZ EMRE. Critical revision of the manuscript for important intellectual content: İnci MERCAN ANNAK, Birgül ERDOĞAN, Nihal YILDIZ EMRE. Study supervision: İnci MERCAN ANNAK, Birgül ERDOĞAN, Nihal YILDIZ EMRE.

## FUNDING INFORMATION

The authors declare that the study received no financial support.

## ETHICS COMMITTEE APPROVAL

Ethical approval was obtained from the Gazi University Ethics Committee for conducting the study under the approval number E‐77082166‐604.01.02‐618792. Regarding the study, the “Voluntary Consent Form” was read to the participants and their verbal consent was obtained. In addition, each participant was assigned a number within the study, and these numbers were used throughout the study and publication process. As a result, participants' personal information remained confidential and was not disclosed to any individual or institution.
